# Prolonged, granulocyte–macrophage colony-stimulating factor-dependent, neutrophil survival following rheumatoid synovial fibroblast activation by IL-17 and TNFalpha

**DOI:** 10.1186/ar2406

**Published:** 2008-04-23

**Authors:** Greg Parsonage, Andrew Filer, Magdalena Bik, Debbie Hardie, Sian Lax, Katherine Howlett, Leigh D Church, Karim Raza, See-Heng Wong, Emily Trebilcock, Dagmar Scheel-Toellner, Mike Salmon, Janet M Lord, Christopher D Buckley

**Affiliations:** 1 Rheumatology Research Group, MRC Centre for Immune Regulation, Institute for Biomedical Research, University of Birmingham, Vincent Drive, Edgbaston, Birmingham B15 2TT, UK

## Abstract

**Introduction:**

A surprising feature of the inflammatory infiltrate in rheumatoid arthritis is the accumulation of neutrophils within synovial fluid and at the pannus cartilage boundary. Recent findings suggest that a distinct subset of IL-17-secreting T-helper cells (T_H_17 cells) plays a key role in connecting the adaptive and innate arms of the immune response and in regulating neutrophil homeostasis. We therefore tested the hypothesis that synovial fibroblasts bridge the biological responses that connect T_H_17 cells to neutrophils by producing neutrophil survival factors following their activation with IL-17.

**Methods:**

IL-17-expressing cells in the rheumatoid synovium, and IL-17-expressing cells in the peripheral blood, and synovial fluid were examined by confocal microscopy and flow cytometry, respectively. Peripheral blood neutrophils were cocultured either with rheumatoid arthritis synovial fibroblasts (RASF) or with conditioned medium from RASF that had been pre-exposed to recombinant human IL-17, TNFα or a combination of the two cytokines. Neutrophils were harvested and stained with the vital mitochondrial dye 3,3'-dihexyloxacarbocyanine iodide before being enumerated by flow cytometry.

**Results:**

T_H_17-expressing CD4^+ ^cells were found to accumulate within rheumatoid synovial tissue and in rheumatoid arthritis synovial fluid. RASF treated with IL-17 and TNFα (RASF_IL-17/TNF_) effectively doubled the functional lifespan of neutrophils in coculture. This was entirely due to soluble factors secreted from the fibroblasts. Specific depletion of granulocyte–macrophage colony-stimulating factor from RASF_IL-17/TNF_-conditioned medium demonstrated that this cytokine accounted for approximately one-half of the neutrophil survival activity. Inhibition of phosphatidylinositol-3-kinase and NF-κB pathways showed a requirement for both signalling pathways in RASF_IL-17/TNF_-mediated neutrophil rescue.

**Conclusion:**

The increased number of neutrophils with an extended lifespan found in the rheumatoid synovial microenvironment is partly accounted for by IL-17 and TNFα activation of synovial fibroblasts. T_H_17-expressing T cells within the rheumatoid synovium are likely to contribute significantly to this effect.

## Introduction

In established rheumatoid arthritis (RA), highly differentiated CD4^+ ^T lymphocytes persist within synovial tissue, and are prevented from undergoing apoptosis by high local concentrations of type I interferons [1]. Simplistically, the preponderance of IFNγ-expressing T cells and the paucity of IL-4-expressing T cells, *in situ *and *ex vivo*, has led to the description of RA as an immune-mediated inflammatory disease that is associated with a predominantly T-helper type-1 T-cell cytokine profile [2-4]. This T-helper type-1 T-cell paradigm, however, does not adequately account for the large numbers of neutrophils that also accumulate within the synovial space.

During active phases of disease, large numbers of activated neutrophils are found in the synovial fluid of both very early RA and established RA patients [5,6]. As a source of proinflammatory mediators such as IL-1β, CXCL8 and TNFα, activated neutrophils clearly contribute to the complex cytokine milieu of the inflamed joint [5]. There is evidence to suggest that CD4 T cells and neutrophils may be engaged in complex cytokine crosstalk. For example the T-helper type-1 T-cell-associated cytokine IL-18 indirectly induces the recruitment of neutrophils in a murine model of arthritis [7]. Similarly, IFNγ-stimulated neutrophils have been shown to release potent chemoattractants for T-helper type-1 T cells and NK cells [8].

Intermittent neutrophil accumulation within the synovial fluid of RA patients results in the degradation of extracellular matrix proteins that are crucial for the lubricative function of synovial fluid. The release of reactive oxygen intermediates and broad-acting proteases from the intracellular granules of neutrophils is responsible for this. Activated neutrophils have also been found at the cartilage pannus interface, where they may promote joint erosion more directly [9]. Furthermore an elegant series of investigations has revealed an essential role for neutrophils (and other cells of the innate immune system) both in the initiation and progression of catastrophic joint inflammation in the murine K/BxN spontaneous arthritis model [10]. Synovial neutrophils are therefore likely to make a significant contribution to the pathology of RA.

We recently described a distinctive but transient synovial fluid cytokine profile in patients with very early synovitis destined to become RA that distinguished such patients from those who did not progress to RA [11]. The T-cell-derived cytokine IL-17 formed part of this distinctive cytokine profile. IL-17 has been implicated in the process of chronic inflammatory pathology at various anatomical locations including the synovium via release from a distinct subset of CD4 T cells termed IL-17-secreting T-helper cells (T_H_17 cells) [12-14].

Consistent with their distinct developmental lineage, which requires the presence of the transcriptional factor retinoic acid-related orphan receptor γt isoform, T_H_17 cells appear to have a unique function in bridging the adaptive and innate arms of the immune system to promote host defence against extracellular bacterial infections [15]. T_H_17 cells also play an important homeostatic role in regulating neutrophil production and blood neutrophil counts [16,17] and in promoting the recruitment of neutrophils into tissues. In addition, recent studies have shown that these cells contribute to bone destruction by activating osteoclasts in models of arthritis [14]. Human T_H_17 cells were described very recently in large numbers in the gut mucosa [18]. In addition, T_H_17 cells have been shown to express the chemokine receptors CCR6 and CCR4 [19] and to depend on IL-1 and IL-6 but not on transforming growth factor beta for their production [20,21].

In the present study we tested the hypothesis that synovial fibroblasts bridge the biological responses that connect the presence of T_H_17 cells in the synovium with the increase in neutrophils seen in RA by producing neutrophil survival factors following their activation with IL-17.

## Materials and methods

### Reagents

All cell culture reagents were endotoxin-free and were purchased from Sigma (St Louis, MO, USA). Recombinant human (rh) granulocyte–macrophage colony-stimulating factor (GM-CSF), rhIL-17A and rhTNFα were from Peprotech (London, UK), monoclonal anti-human GM-CSF (MAB215) and polyclonal anti-human GM-CSF (AF215NA) were from R&D Systems (Abingdon, UK), and recombinant IFNβ was from Biosource (Paisley, UK). Type I IFN receptor (CD118) blocking antibody was obtained from Calbiochem (San Diego, CA), anti-IL-6R and anti-TNFα were from R&D (Abingdon, UK), human GM-CSF and IL-6 ELISA kits were from BDPharmingen (San Diego, CA), and protein G-conjugated agarose was from Upstate (Lake Placid, NY, USA). The 0.2 μm Transwell inserts were obtained from Falcon (BD biosciences, San Jose, CA, USA), rhIL-2 from Chiron (Middlesex, UK), phytohaemaglutinin from Murex (Dartford, UK) and 3,3'-dihexyloxacarbocyanine iodide was from Molecular Probes (Leiden, The Netherlands).

Additional reagents, including adenosine, adenosine deaminase, indomethacin, NS398 and MK886, were from Sigma. The multiplex assay used Beadmate kits (Upstate) as specified in the manufacturer's instructions, read with a Luminex 100 system (Upstate).

### Patient samples, fibroblast culture and treatment

Rheumatoid synovial fibroblasts were isolated from the synovium of RA patients meeting the 1987 American College of Rheumatology criteria [22] undergoing joint replacement surgery. Clinical details of patients fulfilling the 1987 American College of Rheumatology criteria who donated samples are presented in Table 1. The Disease Activity Scores using 28 joint counts (DAS28) at the time of joint replacement are given for patients donating samples from which fibroblasts were cultured. Six of the eight patients undergoing joint replacement who gave samples for fibroblast culture had DAS28 counts >5.2, indicating active disease.

**Table 1:** Clinical details of rheumatoid arthritis patients

Age (years)	Sex	Rheumatoid Factor	Erosive disease	Therapy	DAS28 score
Samples used to culture fibroblasts					
62	Female	+	+	Methotrexate	5.5
52	Female	+	+	Methotrexate	4.4
66	Male	+	+	Etanercept, oral prednisolone	5.5
71	Female	+	+	D-penicillamine	5.3
62	Female	+	+	--	6.8
59	Female	+	+	Methotrexate	6.8
30	Female	+	+	Azathioprine	6.5
77	Male	+	+	Sulphasalazine	4.0
Samples used for confocal microscopy and flow cytometry					
72	Female	+	+	Methotrexate	--
73	Female	+	+	Methotrexate	--
73	Female	+	+	Methotrexate, oral prednisolone	--
62	Female	--	--	Methotrexate, infliximab	--
64	Female	+	+	--	--
66	Female	+	+	Methotrexate	--

Peripheral blood and synovial fluid were also obtained from RA patients meeting the 1987 American College of Rheumatology criteria [22]. Ethical approval for the use of patient-derived material was given by the local research ethics committee, and all patients provided written informed consent (LREC reference 5735).

Fibroblasts were maintained in fibroblast medium (consisting of 81.3% RPMI 1640, 16.3% foetal calf serum, 0.81 × MEM nonessential amino acids, 0.81 mM sodium orthopyruvate, 1.62 mM glutamine, 810 U/ml penicillin and 81 μg/ml streptomycin) at 37°C in a humidified 5% carbon dioxide atmosphere. The fibroblast phenotype was confirmed by morphology and immunofluorescence microscopy as described previously [23]. All cells expressed fibronectin and prolyl-4-hydroxylase, while less than 0.5% cells stained positive for CD68, von Willebrand factor, CD31 or cytokeratin.

Synovial fibroblasts were used between passages 4 and 8. Before the addition of exogenous cytokines, fibroblasts were seeded into flat-bottomed 96-well plates at a density of 6 × 10^3 ^cells/well and were cultured for 48 hours in coculture medium (consisting of 88.9% RPMI 1640 medium supplemented with 8.9% heat-inactivated foetal calf serum, 1.62 mM glutamine, 810 U/ml penicillin, 81 μg/ml streptomycin and 1.8 mM (pH 7.4) HEPES). The coculture medium was then refreshed and the fibroblasts exposed to 10 ng/ml IL-17A and 10 ng/ml TNFα. Following cytokine treatment or coculture, the fibroblasts were washed extensively with RPMI 1640 and were cultured with or without 10^5 ^neutrophils/well for a further 24 hours. Cells or conditioned medium were then harvested.

### Preparation of neutrophils

Peripheral blood of consented healthy volunteers was taken into tubes containing the anticoagulant ethylenediamine tetraacetic acid and was then mixed with T500 Dextran to sediment the red blood cells. Mononuclear cells and granulocytes were separated by centrifugation across a saline-based Percoll gradient (upper phase, 54% vol/vol; lower phase, 79% vol/vol), and were then washed in PBS to remove residual Percoll. Neutrophil preparations were routinely >98% pure and viable, as assessed by May–Grunwald Giemsa-stained cytospins.

### Cell survival assays

Flow cytometry was carried out using the 20 μl volume dump facility of the Beckman Coulter Epics XL bench-top flow cytometer (High Wycombe, UK). When used in combination with vital dyes, this technique enables accurate enumeration of live cells within a sample of defined volume. Neutrophils were gated based on their forward scatter versus side scatter profiles, and apoptotic cells were excluded by loss of 3,3'-dihexyloxacarbocyanine iodide positivity. Survival is expressed as percentage of the starting population. The percentage of cells committed to apoptosis was confirmed with May–Grunwald Giemsa-stained cytospins.

### Cytokine depletion and blockade studies

Supernatants from cytokine-treated fibroblasts, and solutions containing higher concentrations of recombinant target proteins were depleted of cytokine in three rounds of incubation with an excess of irrelevant or depleting antibody-coated protein G-conjugated agarose beads. Residual beads were removed by centrifugation before exposing neutrophils to the medium or performing ELISAs to verify that cytokine depletion was successful (the lower detection limit of the GM-CSF ELISA was 4 pg/ml). For lipopolysaccharide studies, 10 ng/ml lipopolysaccharide (Sigma) was added to neutrophils in the presence or absence of conditioned medium from IL-17A-treated and TNFα-treated cells and of 50 μg/ml polymyxin B sulphate.

### Inhibitor studies

Freshly isolated neutrophils were pretreated with Ly294002 (20 μM) or Bay 11-7085 (1 μM) or vehicle control for 45 minutes at 37°C, washed, and cultured for 24 hours in fibroblast-conditioned medium (FCM) from untreated rheumatoid arthritis synovial fibroblasts (RASF) or from rheumatoid arthritis synovial fibroblasts stimulated with TNFα and IL-17 (RASF_IL-17/TNF_). The optimal concentration of inhibitors used was established by performing a dose–response analysis.

### Neutrophil functional studies

To measure the functionality of neutrophils, superoxide release upon stimulation with 200 nM f-Met-Leu-Phe was measured using the substrate cytochrome c. Following their 24-hour treatments, neutrophils were washed once in HBSS buffer (HBSS pH 7.3 + 25 mM HEPES and 5 mM glucose), and were resuspended in HBSS containing 1% human serum. Then 5 × 10^4 ^neutrophils were added to triplicate wells of a 96-well plate in the presence or absence of f-Met-Leu-Phe. Release of superoxide was quantified by measuring the colour change of cytochrome c at 450 nm emission. The positive control was complete reduction of cytochrome c using the reducing agent sodium dithionite (100 mM).

### Inhibitor of NFκB measurement

Degradation of inhibitor of NFκB in the presence of 10 ng/ml TNFα or FCM was assessed by western blotting. Neutrophils (at least 5 million cells/sample) were lysed with 10% trichloroacetic acid (Sigma) followed by 5 minutes incubation on ice. The lysates were centrifuged for 5 minutes at 14,000 × *g *at 4°C and the supernatant was discarded. The pellet was washed twice with ice-cold acetone, centrifuging each time as before. The pellet was dissolved in SDS-PAGE sample loading buffer (0.125 M Tris pH 6.8 containing 20% glycerol, 2% SDS, 5% mercaptoethanol and 25 μg/ml bromophenol blue). The sample was boiled, then cooled on ice for 5 minutes each, before performing gel electrophoresis using a 12% SDS gel.

Proteins were then transferred to a polyvinylidene difluoride membrane (Flowgen Ltd, Sittingbourne, UK) for 2 hours at 0.45 A using a wet blotting system (Biorad Hemel Hempstead, UK). The blots were blocked for 1 hour with 5% skimmed milk in Tris-buffered saline containing 0.1% Tween 20 (TBST) for 1 hour. Blots were incubated with primary antibodies (IkBα Rabbit IgG; Upstate) diluted in Tris-buffered saline containing 1% skimmed milk overnight at 4°C, washed four times with TBST for 10 minutes each, and then incubated with secondary antibodies (horseradish peroxidase-donkey anti-rabbit; Amersham, Bucks, UK) diluted in TBST. Blots were washed three times with TBST for 10 minutes each and developed for 5 minutes with enhanced chemiluminescence reagents (Amersham).

### Flow cytometry analysis of peripheral blood and synovial fluid cells for IL-17 expression

Peripheral blood mononuclear cells and synovial fluid mononuclear cells were isolated by density gradient centrifugation on Ficoll-paque™-Plus (GE Healthcare, Slough, UK). Following centrifugation, peripheral blood mononuclear cells and synovial fluid mononuclear cells were isolated from the buffy coat and were added to fresh RPMI 1640 medium. Cells were washed twice with RPMI 1640 medium by centrifugation at 600 × *g *for 8 minutes. Cells were then counted and resuspended at 1 × 10^6 ^cells/ml in fresh medium. Intracellular IL-17 was detected by flow cytometry after stimulation of cells with 50 ng/ml phorbol 12-myristate 13-acetate (PMA) (Sigma) and 250 ng/ml ionomycin (Sigma) in the presence of 10 μg/ml Breveldin A (Sigma) for 4 hours in a 24-well plate (Sarstedt, Leicester, UK).

Cells were removed from culture and resuspended in 2% BSA/PBS. Cell surface staining with fluorescein isothyocyanate (FITC)-labelled anti-CD4 (Immunotools, Friesoythe, Germany), phycoerythrin-cyanine 5-labelled anti-CD3 (Immunotools), FITC-labelled IgG_1 _isotype control (Immunotools), and phycoerythrin-cyanine 5-labelled IgG_1 _isotype control (Serotec, Oxfordshire, UK) was performed by incubating cells on ice with the appropriate antibody combination for 20 minutes. Cells were subsequently fixed and permeabilised with Cytoperm according to manufacturer's instructions (Caltag/Invitrogen, Towcester, UK).

Cells were incubated with either phycoerythrin-labelled anti-IL-17 (eBiosciences, San Diego, CA, USA) or phycoerythrin-labelled IgG_1 _isotype control (Immunotools) for 20 minutes, and were then washed and acquired on a Beckman Coulter Epics XL bench-top flow cytometer. T cells were gated based on their forward scatter versus side scatter profiles and their CD3 expression. CD4^+ ^IL-17^+ ^T cells are expressed as a percentage of the CD3^+ ^population.

### Confocal microscopy

Full thickness (5 μm) sections of human rheumatoid synovium were prepared and fixed in 100% acetone at 4°C for 20 minutes. The sections were rehydrated with PBS pH 7.4 with 5% foetal calf serum (Biosera Ltd, Ringmer, UK), and indirect immunofluorescence was performed using the following primary antibody combinations: IgG_2b _mouse anti-human CD3 (UCHT-1, 17 μg/ml; gift from Peter Beverley, University College Hospital, London, UK) with rabbit anti-human von Willibrand factor (A0082, 6.2 μg/ml; Dako, Glostrup, Denmark) and IgG_1 _mouse anti-human IL-17 (12-7179, 1.25 μg/ml; eBiosciences); or IgG_2b _mouse anti-human CD4 (OKT4) with IgG_2a _mouse anti-human CD8 (OKT8, both OKT clones used as ascitic fluid 1/100; American Type Culture Collection, Middlesex, UK) and rabbit anti-human IL-17 (AHP455G, 20 μg/ml; AbD Serotec, Oxfordshire, UK).

The secondary antibody combinations used were: goat anti-mouse IgG_2b_-cyanine 5 (1090-15, 4 μg/ml; Southern Biotech Inc., Birmingham, AL, USA) with donkey anti-rabbit-tetramethyl-rhodamine (711-026-152, 6 μg/ml; Jackson Immuno-Research, Newmarket, UK) and goat anti-mouse IgG_1_-FITC (1070-02, 20 μg/ml; Southern Biotech Inc.); or goat anti-mouse IgG_2b_-cyanine 5 (1090-15, 4 μg/ml; Southern Biotech Inc.) with goat anti-mouse IgG_2a_-FITC (1080-02, 20 μg/ml; Southern Biotech Inc.) and donkey anti-rabbit-tetramethyl-rhodamine (711-026-152 6 μg/ml; Jackson Immuno-Research).

Irrelevant control antibodies (X0931 and X0903; Dako) were used to confirm IL-17 staining, while primary antibodies were omitted as controls for other stains. Sections were counterstained with nuclear DNA dye Hoechst 33258 (20 μg/ml; Riedel De Haenag, Seelze, Hannover, Germany) and were subsequently mounted in 2.4% 1,4-diazabicyclo [2.2.2]octane (Aldrich, Gillingham, UK) in glycerol pH 8.6 (Fisons Scientific, Loughborough, UK). A Zeiss 510 laser-scanning confocal microscope (Zeiss, Welwyn Garden City, UK) was used to visualise staining with images captured and processed using the Zeiss LSM Image Examiner software (Zeiss).

### Statistical analysis

A nonparametric distribution was assumed for all assays. Statistical analysis of differences in neutrophil survival induced by fibroblasts or FCM across multiple groups was performed using Kruskal–Wallis one-way analysis of variance and Dunn's post test. For the difference between paired samples (depletions, blocking experiments, experiments with and without transwells), significance was assessed using the Wilcoxon signed rank test with two-tailed *P *values. Results are presented as the mean ± standard deviation or standard error where appropriate.

## Results

### IL-17-expressing CD4 T cells accumulate within the rheumatoid synovium

**Figure 1:**
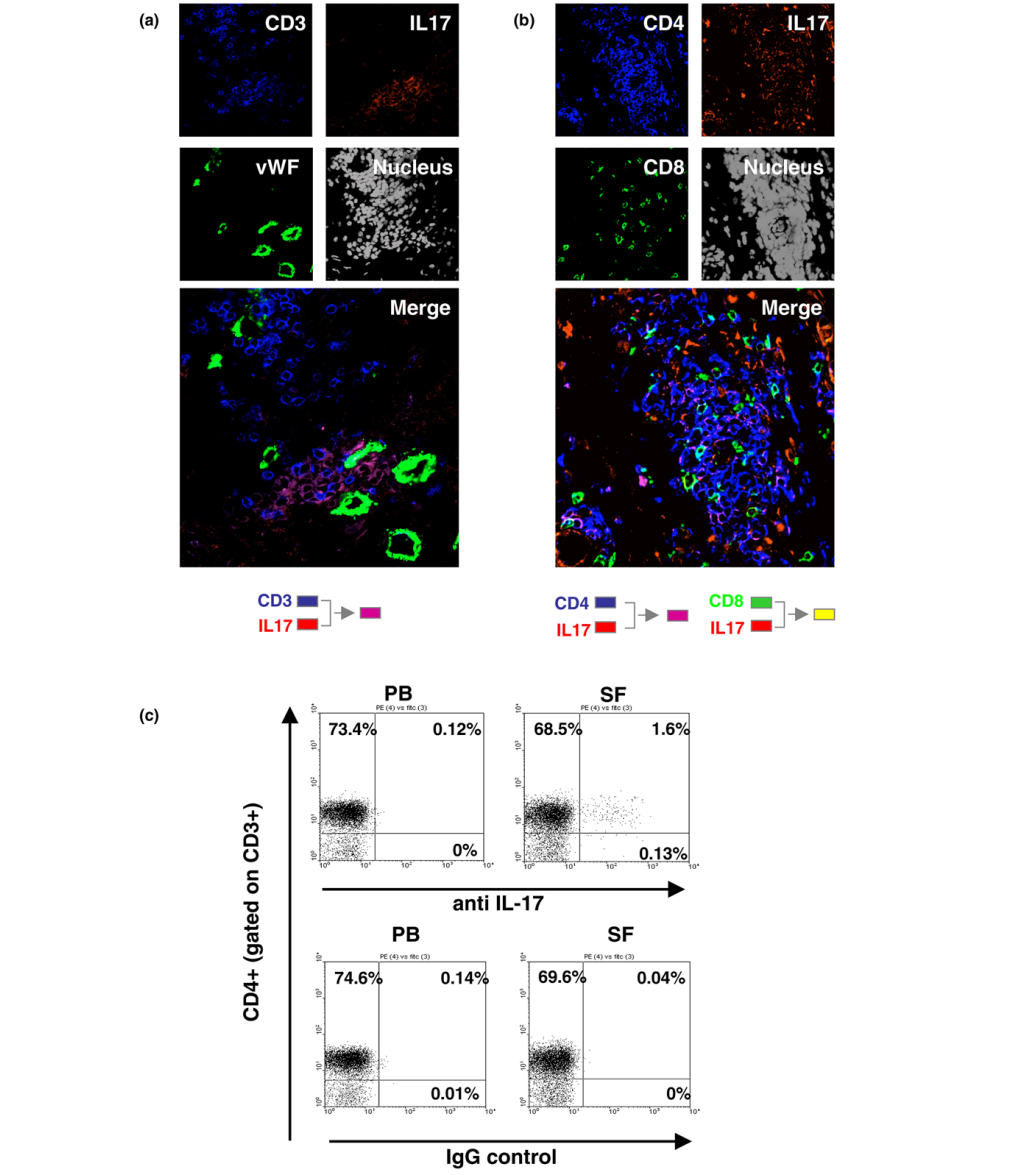
**IL-17 expression on T cells within rheumatoid synovium and in synovial fluid CD4^+^CD3^+ ^T cells**. **(a) **Rheumatoid synovial tissue was examined by immunohistochemistry. IL-17 (red) was found to colocalise with CD3^+ ^T cells (blue) in perivascular cuffs (purple). Blood vessels were localised with von Willebrand factor (vWF) (green). Nuclear staining is shown in grey. **(b) **IL-17 (red) expression is associated with CD4^+ ^T cells (blue) but not CD8^+ ^(green) T cells. Nuclear staining is shown in grey. **(c) **flow cytometric analysis of peripheral blood (PB) and synovial fluid (SF) CD3^+ ^T cells demonstrates that IL-17 is expressed in SF CD4^+ ^T cells. PE, phycoerythrin; FITC, fluorescein isothyocyanate.

We first determined whether T_H_17 cells are present within the rheumatoid synovium. IL-17 was expressed on CD3^+ ^T cells within the synovium, and in particular on T cells found in a perivascular distribution (Figure 1a). Expression of IL-17 was confined to CD4 T cells, with no expression on CD8 T cells, although there were some IL-17-expressing cells in the synovium that did not express CD4 or CD8 (Figure 1b). Equally some CD4 T cells did not express IL-17 (Figure 1a,b). IL-17-expressing CD4^+^CD3^+ ^cells were also found at low frequency within rheumatoid synovial fluid (Figure 1c).

### IL-17 and TNFα synergise to significantly enhance fibroblast-mediated neutrophil survival

Previous studies have shown that IL-17 and TNFα synergise with each other to produce potent biological effects in leucocytes. We therefore cross-titrated recombinant human IL-17A and TNFα on RASF to determine whether these cytokines, either individually or in combination, could enhance the ability of RASF to support neutrophil viability in cocultures of neutrophils with fibroblasts.

**Figure 2:**
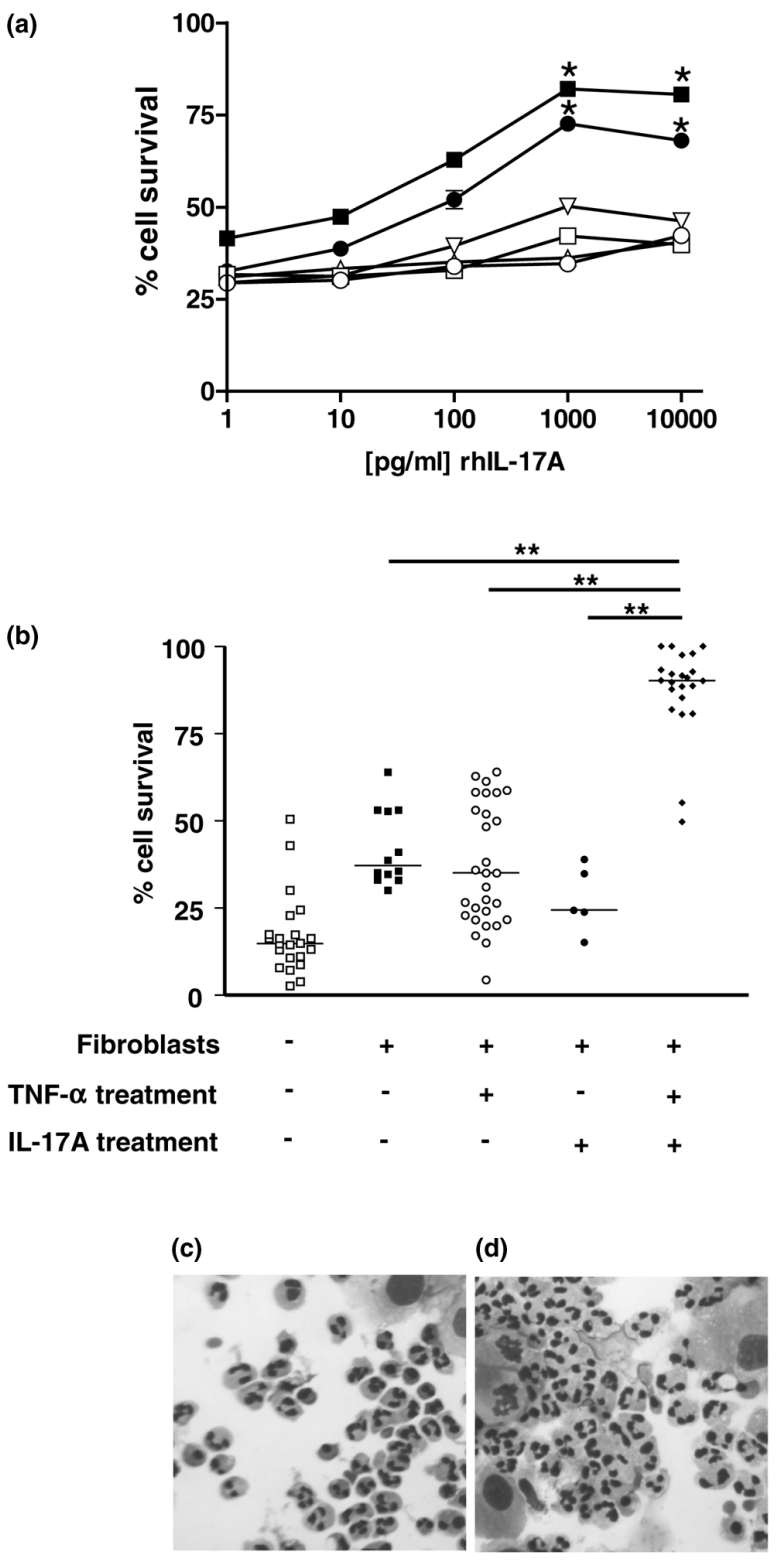
**Rheumatoid arthritis synovial fibroblasts stimulated with TNFα and IL-17 efficiently extend neutrophil survival**. **(a) **Peripheral blood neutrophils were cocultured with rheumatoid arthritis synovial fibroblasts (RASF) pretreated for 24 hours with the indicated concentrations of cytokines. Recombinant human (rh)TNFα concentrations: open circle, 0 pg/ml; open square, 1 pg/ml; open triangle, 10 pg/ml; open inverted triangle, 100 pg/ml; filled circle, 1,000 pg/ml; filled square, 10,000 pg/ml. *P < 0.05 versus rhTNFα = 0 pg/ml. **(b) **Peripheral blood neutrophils were cultured alone or cocultured with RASF pretreated for 24 hours with the indicated cytokines both at a concentration of 10 ng/ml. **P < 0.01. Data represent mean ± standard deviation from at least five independent experiments. Absolute neutrophil survival was determined by flow cytometry using fixed volume dumping, with exclusion of apoptotic cells by gating on cells with a maintained mitochondrial membrane potential as assessed by 3,3'-dihexyloxacarbocyanine iodide staining. Neutrophil morphology was examined on cytospins after 24 hours of coculture with **(c) **untreated RASF or **(d) **RASF stimulated with TNFα and IL-17.

Figure 2a shows that the basal level of neutrophil survival in the presence of RASF increases significantly and in a dose-dependent manner when RASF are pretreated with a combination of IL-17 and TNFα. As would be expected, the increase in survival is mirrored precisely by an inhibition of apoptosis (data not shown). Optimal neutrophil survival was achieved at a dose of 1 to 10 ng/ml IL-17 and 10 ng/ml TNFα. Pretreatment of RASF with the highest doses (10 ng/ml) of IL-17 or TNFα alone did not significantly enhance survival at 24 hours (Figure 2b). Neutrophils cocultured with fibroblasts that had been pretreated with IL-17 and TNFα (RASF_IL-17/TNF_), however, remained viable for 24 hours. Indeed, the level of viability exceeded that obtained with a 100 ng/ml dose of rhGM-CSF – a prototypical neutrophil survival factor added directly to neutrophil monoculture (data not shown). Direct microscopic analysis of fibroblast-mediated neutrophil survival by cellular morphology and active caspase-3 immunostaining confirmed these results (Figure 2c,d and data not shown).

### Coculture with RASF_IL-17/TNF _doubles the functional lifespan of neutrophils

**Figure 3:**
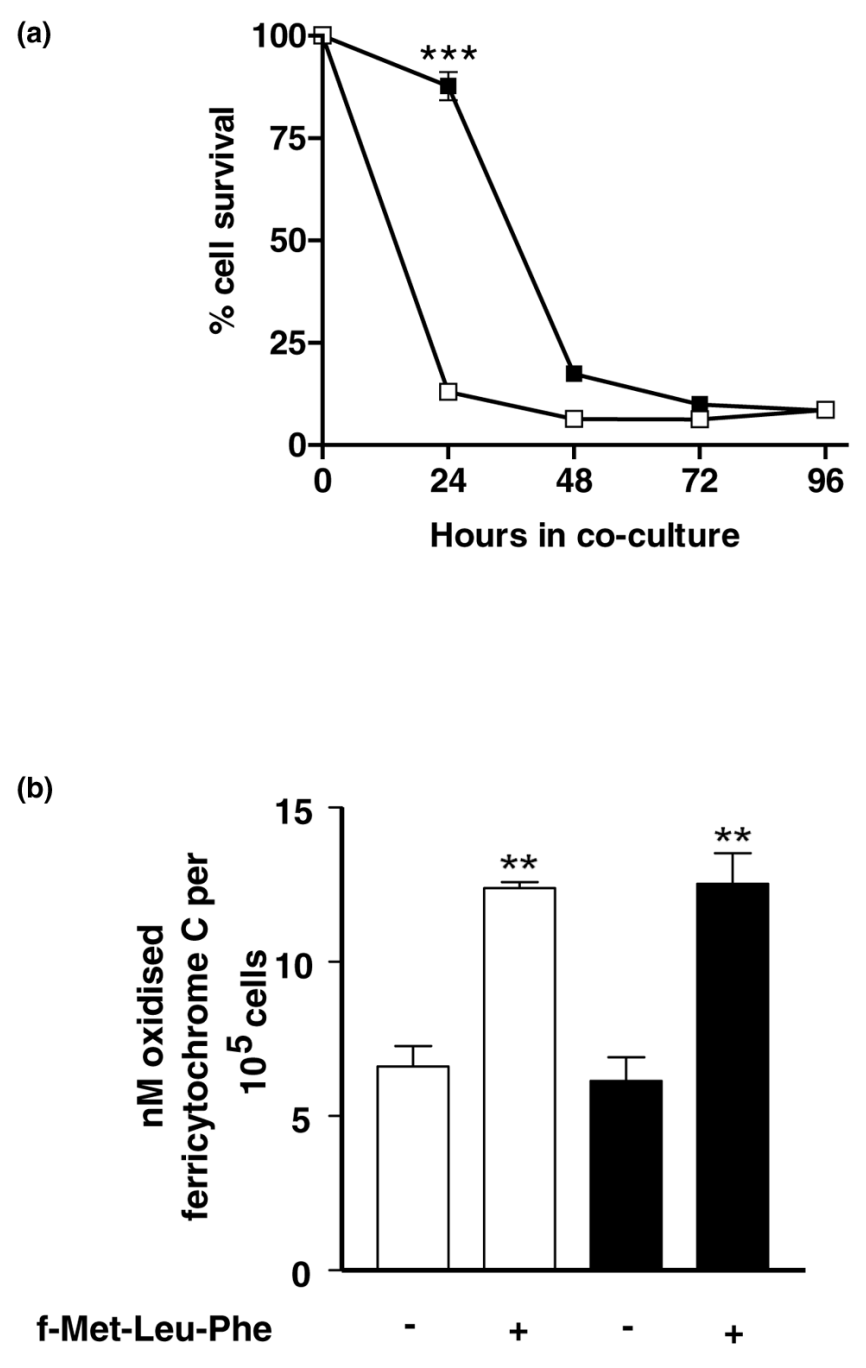
**Coculture with stimulated rheumatoid arthritis synovial fibroblasts doubles the functional lifespan of peripheral blood neutrophils**. **(a) **Peripheral blood neutrophils were cocultured with untreated rheumatoid arthritis synovial fibroblasts (RASF) (open squares) or with RASF stimulated with IL-17 and TNFα (RASF_IL-17/TNF_) (closed squares), and their survival was assessed every 24 hours by flow cytometry. ***P < 0.001. **(b) **The ability to produce superoxide radical in response to f-Met-Leu-Phe was determined in freshly isolated neutrophils (open bars) and neutrophils cocultured with RASF_IL-17/TNF _for 24 hours (filled bars). **P < 0.01 versus unstimulated cells.

We next determined the kinetics of rescue from apoptosis, mediated by coculture of neutrophils with RASF_IL-17/TNF_. Figure 3a shows that, in the presence of RASF_IL-17/TNF_, the neutrophil lifespan is effectively doubled. Furthermore, rescued neutrophils were functionally active and capable of generating the superoxide radical to the same extent as freshly isolated neutrophils upon treatment with f-Met-Leu-Phe (Figure 3b).

### Delayed neutrophil apoptosis is attributable to soluble, temperature-sensitive factors secreted by RASF_IL-17/TNF_

**Figure 4:**
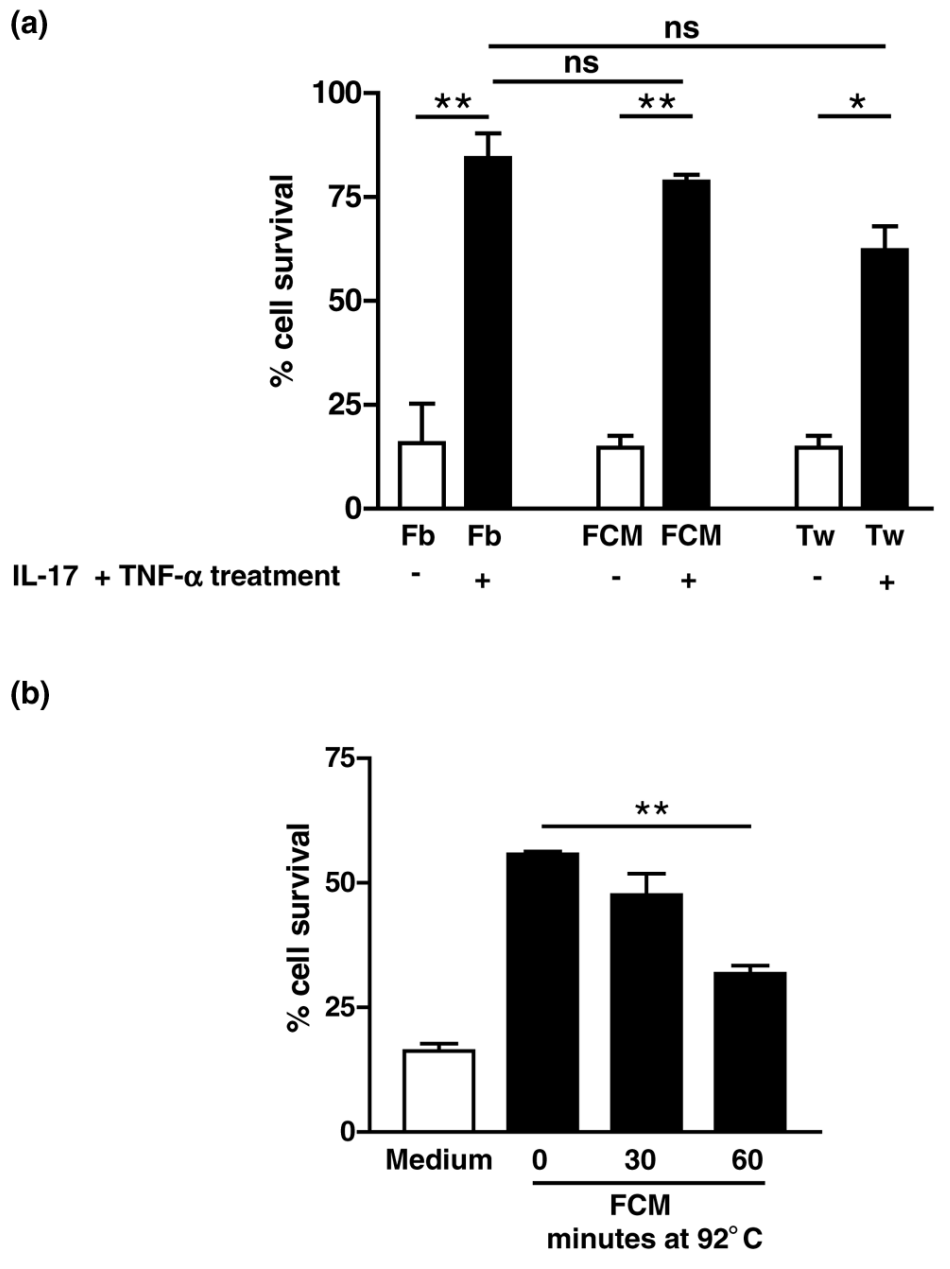
**Soluble, temperature-sensitive factors released by stimulated rheumatoid arthritis synovial fibroblasts extend neutrophil survival**. **(a) **Peripheral blood neutrophils were either cocultured with fibroblasts (Fb), with conditioned medium from IL-17 and TNFα pretreated fibroblasts (FCM), or on a transwell filter suspended above fibroblasts (Tw) for 24 hours. Error bars show the mean ± standard deviation from three independent experiments. **P < 0.01, *P < 0.05; ns, nonsignificant. **(b) **Culture supernatant from rheumatoid arthritis synovial fibroblasts stimulated with IL-17 and TNFα was heated to 92°C for the times indicted before culture with neutrophils for 24 hours, and neutrophil survival was measured. Error bars show the mean ± standard deviation from three independent experiments

We next investigated the mechanism by which cytokine-treated synovial fibroblasts prolonged neutrophil survival by comparing the ability of FCM and transwell-separated neutrophil–synovial cocultures to influence neutrophil survival (Figure 4a). In addition to exposing neutrophils to the high local concentrations of cytokines released from fibroblasts, coculture allows direct cell–cell and cell–matrix interactions to occur. Separating neutrophils from fibroblasts by a transwell filter or using FCM prohibits direct cell contact between the two cell types, but allows soluble factors to be shared.

We observed no significant differences between the rescue afforded by direct coculture, FCM or transwell-separated cultures, indicating that soluble factors released from RASF_IL-17/TNF _are both necessary and sufficient to extend the functional lifespan of neutrophils in cocultures with activated synovial fibroblasts. Furthermore, at least 50% of the survival effect mediated by the soluble factor was temperature sensitive as activity was destroyed following the treatment of conditioned medium at 92°C (Figure 4b).

### GM-CSF partially accounts for neutrophil rescue by cytokine-activated fibroblasts and CD4 T-cell-RASF cocultures

In an attempt to identify which soluble factor(s) might be responsible for neutrophil survival, we screened the FCM from RASF_IL-17/TNF _for a range of cytokines and chemokines using multiplex bead ELISAs. This screening revealed elevated levels of GM-CSF, granulocyte colony-stimulating factor, CCL2 and CXCL8 in FCM from RASF_IL-17A/TNF _compared with untreated RASF or with RASF treated with IL-17 or TNF alone (Table 1).

**Figure 5:**
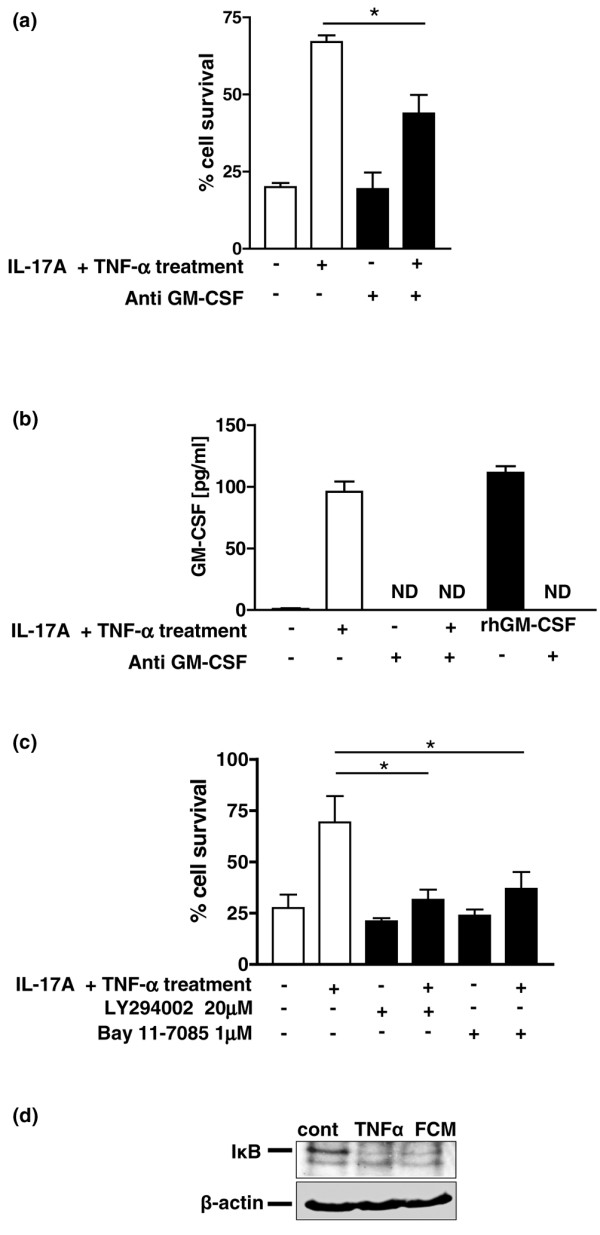
**GM-CSF in conditioned medium from stimulated rheumatoid arthritis synovial fibroblasts maintains neutrophil viability**. Conditioned medium from rheumatoid arthritis synovial fibroblasts stimulated with IL-17 and TNFα (RASF_IL-17/TNF_) maintains neutrophil viability in part through the release of granulocyte–macrophage colony-stimulating factor (GM-CSF) and via phosphatidylinositol-3-kinase-dependent and NF-κB-dependent pathways. **(a) **Using either an irrelevant control antibody (open bars) or specific GM-CSF antibodies (filled bars) conjugated to agarose beads, serum-free conditioned medium (unstimulated or IL-17A/TNFα stimulated) was depleted of GM-CSF and added to freshly isolated peripheral blood neutrophils for 24 hours. Error bars show the mean ± standard deviation from three independent experiments. *P < 0.05. **(b) **The degree of depletion of GM-CSF was determined by ELISA in fibroblast-conditioned medium (FCM) from unstimulated or IL-17A/TNFα-stimulated FCM, before (open bars) and after (filled bars) depletion with anti-GM-CSF antibodies/agarose beads. A fixed dose of 100 pg/ml recombinant human (rh)GM-CSF was used as a positive control for the ELISA and to check the efficiency of GM-CSF depletion (filled bars). ND, not detectable. **(c) **Freshly isolated neutrophils were pretreated with vehicle control (open bars), 20 μM Ly294002 (filled bars) or 1 μM Bay 11-7085 (filled bars) before being cultured for 24 hours in FCM from unstimulated or IL-17/TNFα-stimulated fibroblasts. *P < 0.05. **(d) **Neutrophils that had been exposed to medium alone (cont), TNFα (as a positive control), or IL-17/TNFα-stimulated FCM were subjected to western blotting and were labelled using primary antibodies to inhibitor of NF-κB (IκB) and, as a loading control, β-actin.

Since GM-CSF is a well-characterised neutrophil survival factor, we tested whether this was the factor responsible for neutrophil survival by specific immunodepletion from FCM using depleting antibodies. We consistently detected approximately 100 pg/ml GM-CSF in FCM from RASF_IL-17/TNF_. Following antibody-mediated depletion, the GM-CSF concentrations fell to levels that were essentially undetectable by ELISA (Figure 5b). We found that GM-CSF only accounted for approximately 50% of the neutrophil rescue activity released by synovial fibroblasts in response to IL-17 and TNFα (Figure 5a). Combined immunodepletion of GM-CSF and other candidate survival factors detected in FCM from RASF_IL-17/TNF _– such as granulocyte colony-stimulating factor, CCL2 and CXCL8 – failed to inhibit neutrophil survival any further.

Using an inhibitor of the phosphatidylinositol-3-kinase signalling pathway (Ly294002) and an inhibitor of the NF-κB pathway (Bay11-7085), we found that both phosphatidylinositol-3-kinase and NF-κB signalling pathways contributed significantly to the RASF_IL-17/TNF_-mediated neutrophil survival – implying that in addition to GM-CSF, another factor that utilises phosphatidylinositol-3-kinase and/or NF-κB also plays a role (Figure 5b). We confirmed the role of the NF-κB pathway by demonstrating inhibitor of NF-κB (I-κB) degradation on a western blot of cell lysates from neutrophils treated with FCM from RASF_IL-17/TNF _(Figure 5d).

**Figure 6:**
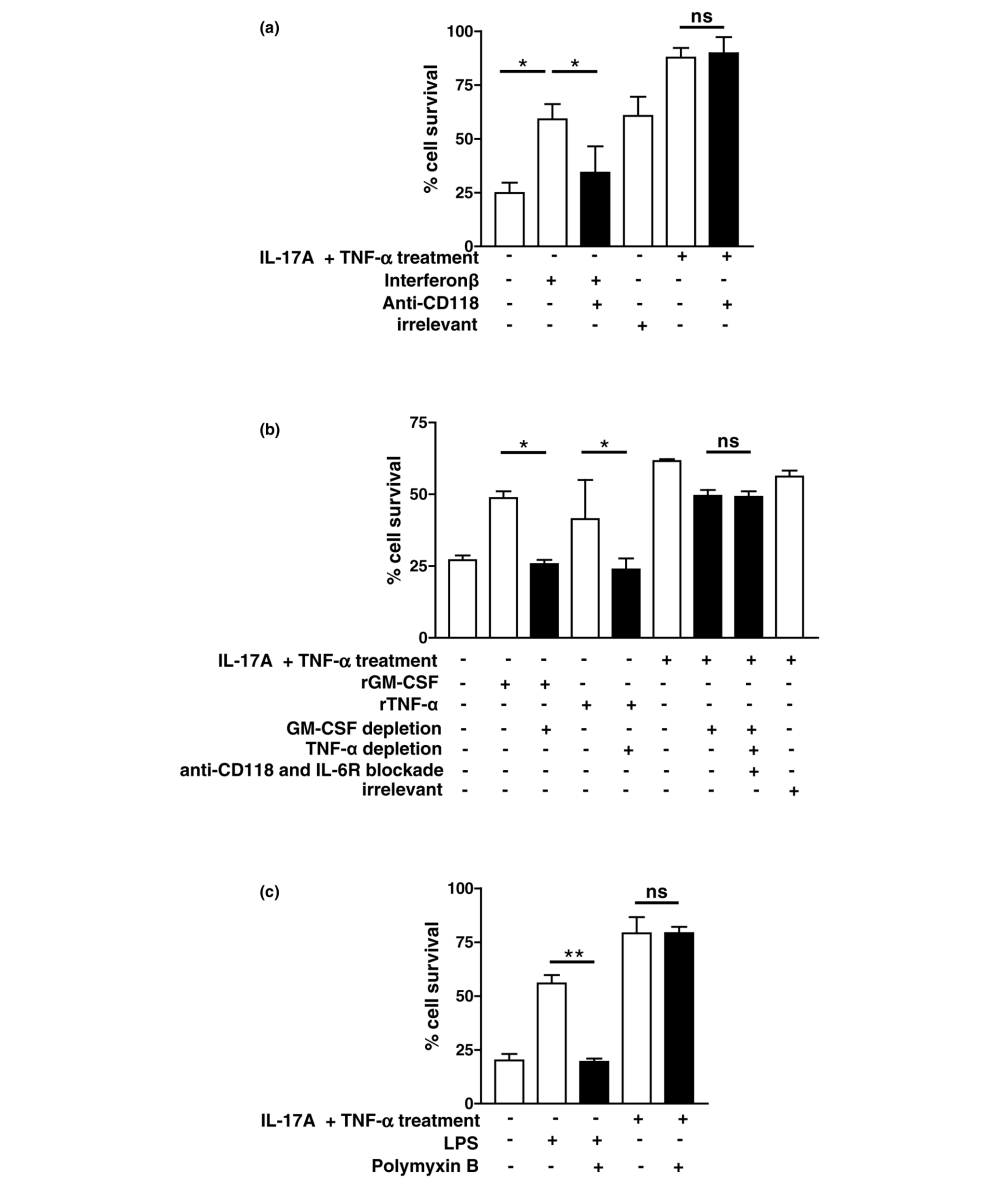
**Lack of contribution of IFNβ, TNFα, IL-6 and lipopolysaccharide to survival induced by conditioned medium**. **(a) **Recombinant IFNβ or fibroblast-conditioned medium (FCM) from rheumatoid arthritis synovial fibroblasts stimulated with TNFα and IL-17 (RASF_IL-17/TNF_) were added to neutrophils in the presence or absence of an anti-CD118 (type I interferon receptor) blocking antibody (filled bars) or irrelevant control. **(b) **Using either irrelevant control antibodies, specific granulocyte–macrophage colony-stimulating factor (GM-CSF) and/or TNFα antibodies conjugated to agarose beads, serum-free conditioned medium (unstimulated or IL-17A/TNFα stimulated) was depleted of GM-CSF and/or TNFα and added to freshly isolated peripheral blood neutrophils for 24 hours. In some experiments, additional blockade of IFNβ receptors (CD118) and IL-6 receptors was employed after depletion steps. Error bars show the mean ± standard deviation from three independent experiments. **(c) **Lipopolysaccharide (10 ng/ml) or FCM from RASF_IL-17/TNF _was added to neutrophils in the presence or absence of polymyxin B (50 μg/ml, filled bars). **P < 0.01, *P < 0.05; ns, nonsignificant.

Involvement of NF-κB suggested further possible neutrophil survival candidates, including TNFα, IL-6 and IFNβ. No TNFα was present, however, in the multiplex assay of cytokine-pretreated fibroblast supernatants (Table 2). IL-6 is produced in large quantities by activated fibroblasts (Table 2). IFNβ is a well described, fibroblast-derived neutrophil survival factor. Using an effective blocking antibody to the IFNβ receptor, we showed that blockade of IFNβ within neutrophil survival experiments had no effect on the enhanced survival seen in the presence of conditioned medium (Figure 6a). We found that recombinant IL-6 does not induce a significant delay in neutrophil apoptosis in this system (data not shown), On the basis that synergy between multiple survival candidates could be leading to enhanced survival, however, we performed a combined experiment in which GM-CSF and TNFα were depleted from conditioned media, before adding to neutrophils pretreated with blocking antibodies to interferon and IL-6 receptors (Figure 6b). Although recombinant TNFα induced some survival, the combined effect of all depletions and blockade was no greater than the effect of GM-CSF blockade alone.

**Table 2:** Cytokine and stromal factor levels in cell culture supernatants

Analyte	Analyte concentration			
	
	Medium alone	TNFα	IL-17A	IL-17A and TNFα
IL-1β	--	--	--	--
IL-2	--	--	--	--
IL-6	*****	******	******	*******
IL-7	--	*	--	*
CXCL8	--	*****	--	******
IL-10	--	--	--	--
IL-15	--	--	--	--
Granulocyte–macrophage colony-stimulating factor	--	*	--	**
Granulocyte colony-stimulating factor	*	**	*	*****
CCL2	--	--	--	*****
CCL3	*	***	*	*
CCL5	--	***	--	*
CXCL10	*	*	*	*
TNFα	--	--	--	--

Supernatant from rheumatoid arthritis synovial fibroblasts cultured in medium alone, IL-17A, TNFα or both IL-17 and TNFα were assayed by multiplex ELISA for cytokines and chemokines, and by ELISA for IL-6 (lower detection limit, 15 pg/ml). Duplicate wells for each sample were analysed and the data shown are after subtracting background levels from wells. --, undetectable and <1 pg/ml; *, 1 to 50 pg/ml; **, 50.1 to 100 pg/ml; ***, 100.1 to 500 pg/ml; ****, 500.1 to 1,000 pg/ml; *****, 1,000.1 to 5,000 pg/ml; ******, 5,000.1 to 10,000 pg/ml; *******, 10,000.1 to 50,000 pg/ml.

We hypothesised that the temperature-insensitive component of neutrophil rescue might result from the presence of adenosine, arachidonic acid derivatives, or contaminating lipopolysaccharide. Neither adenosine nor adenosine deaminase, however, affected neutrophil survival. Furthermore, neither the cyclooxygenase-2 inhibitors indomethacin and NS398 nor the 5-lipooxygenase inhibitor MK-886 inhibited survival induced by FCM from RASF_IL-17/TNF _(data not shown). To rule out an effect of contaminating lipopolysaccharide, we used polymyxin B to bind lipopolysaccharide, but this did not inhibit neutrophil rescue (Figure 6c).

## Discussion

We have previously shown that IL-17 can be detected by multiplex-bead ELISA in the synovial fluid of patients with early synovitis destined to develop RA [11]. Here we show that an important biological consequence of IL-17, produced by CD4 T_H_17 cells found in the rheumatoid synovium, is enhanced neutrophil survival. This survival effect resulting from inhibition of spontaneous neutrophil apoptosis is mediated in part by synovial fibroblast-derived GM-CSF. When stimulated with IL-17 and TNFα, synovial fibroblasts produced soluble survival factors that effectively doubled the functional lifespan of neutrophils. This activity was significantly reduced by pretreatment of neutrophils with the phosphatidylinositol-3-kinase inhibitor Ly294002 and the NF-κB inhibitor Bay 11-7085. Our findings demonstrate that T_H_17 cells are found in the rheumatoid synovium, and extend the observations of T_H_17 cells in mice models of autoimmune arthritis to human RA.

Other studies have measured a slight increase in GM-CSF and IL-6 secretion from human bronchial epithelial cells, human umbilical vein endothelial cells and RASF in response to IL-17 treatment alone [24,25]. We, however, observed no reproducible enhancement of RASF-mediated neutrophil survival after pretreatment with IL-17 alone. This suggests that, at least in the rheumatoid synovium, there appears to be a stringent requirement for cytokine synergism (IL-17 and TNFα) to produce functionally relevant levels of neutrophil survival factors. Together with recent data suggesting a role for IFNγ in the resolution phase of inflammation [26], the description of a role for T_H_17 cells in murine models of arthritis, and our finding of T_H_17 cells in the rheumatoid synovium, this raises the question of how useful it is to view RA as a T-helper type-1 T-cell-associated pathology.

Laan and coworkers found that systemic administration of a function-blocking anti-GM-CSF antibody to mice prevented the accumulation of neutrophils in bronchoalveolar fluid following intranasal treatment with IL-17A and TNFα [24]. The authors did not attribute this effect to blockade of neutrophil survival in lung tissues, but to blockade of granulopoiesis. Interestingly it has been reported that a RA patient receiving rhGM-CSF in order to treat concomitant agranulocytosis (Felty's syndrome) suffered a flare in arthritis as a direct result of treatment [27]. Our data suggest that stromal cell-derived GM-CSF is likely to be important for IL-17 and TNFα-induced neutrophil survival at sites of inflammation, including the RA synovium.

It is clear that other unknown factors within FCM are also required to achieve efficient prolongation of neutrophil survival. We have also eliminated granulocyte colony-stimulating factor as a candidate for neutrophil survival, despite detecting very high concentrations of this protein in the supernatant from IL-17 and TNFα-stimulated fibroblasts. This elimination is consistent with the fact that granulocyte colony-stimulating factor acts at an earlier stage to promote granulopoiesis [28], whereas GM-CSF has an effect both to promote granulopoiesis in the bone marrow and to prevent neutrophil apoptosis in tissues. We also detected very high concentrations of the neutrophil chemokine CXCL8 in FCM. We observed that neutrophils in coculture with RASF exhibited a highly motile phenotype, yet blockade of CXCL8 had no effect on neutrophil survival (data not shown). In this context, CXCL8 is reported to have a more significant role in the recruitment and priming of neutrophils than in their protection from apoptosis, especially in the context of GM-CSF-mediated rescue [24,29].

## Conclusion

Taken together, our data suggest that the presence of CD4^+ ^T_H_17 cells and TNFα is capable of perpetuating a neutrophil infiltrate through an interaction with synovial fibroblasts within the rheumatoid synovium. Granulocyte colony-stimulating factor, and perhaps GM-CSF acting systemically to promote granulopoiesis, combined with local release and endothelial presentation of CXCL8 may be responsible for increasing the production and release of neutrophils from the bone marrow and their subsequent recruitment to inflamed tissues. Once neutrophils arrive in the tissue, however, we propose that local production of GM-CSF, by IL-17A and TNFα-stimulated fibroblasts, prolongs their functional lifespan. The cytokine profiles in synovial fluid from early RA patients and established RA patients are therefore consistent with a microenvironment that contains freely diffusible survival factors for neutrophils, produced by synovial fibroblasts. Our findings provide a potential molecular explanation for the persistently high levels of neutrophils found in the inflamed rheumatoid microenvironment, by linking T_H_17 cells to neutrophil survival via synovial fibroblasts.

## Abbreviations

BSA = bovine serum albumin; ELISA = enzyme-linked immunosorbent assay; FCM = fibroblast-conditioned medium; FITC = fluorescein isothyocyanate; GM-CSF = granulocyte–macrophage colony-stimulating factor; HBSS = Hank's Buffered saline solution; IL = interleukin; IFN = interferon; MEM = Eagle's "Minimal Essential Media"; NF = nuclear factor; PBS = phosphate-buffered saline; RA = rheumatoid arthritis; RASF = rheumatoid arthritis synovial fibroblasts; RASF_IL-17/TNF _= rheumatoid arthritis synovial fibroblasts stimulated with TNFα and IL-17; rh = recombinant human; TBST = Tris-buffered saline containing 0.1%; Tween 20; T_H_17 cells = IL-17-secreting T-helper cells; TNF = transforming growth factor.

## Competing interests

The authors declare that they have no competing interests.

## Authors' contributions

GP and AF contributed equally to this work. GP and AF conceived of the study, participated in its design and coordination, carried out the coculture experiments, and helped to draft the manuscript. DH and SL carried out confocal microscopy. LDC performed flow cytometry of IL-17 cells. MB, KH, ET and S-HW performed coculture and inhibitor experiments. DS-T, KR, MS and JML participated in the study design and coordination, and revised the manuscript critically. CDB conceived of the study, participated in its design and coordination, and helped to draft the manuscript. All authors read and approved the final manuscript.
